# Distinct phenotypes of new transmembrane-domain neuregulin 1 mutant mice and the rescue effects of valproate on the observed schizophrenia-related cognitive deficits

**DOI:** 10.3389/fnbeh.2014.00126

**Published:** 2014-04-14

**Authors:** Ju-Chun Pei, Chih-Min Liu, Wen-Sung Lai

**Affiliations:** ^1^Laboratory of Integrated Neuroscience and Ethology, Department of Psychology, National Taiwan UniversityTaipei, Taiwan; ^2^Department of Psychiatry, National Taiwan University HospitalTaipei, Taiwan; ^3^Graduate Institute of Brain and Mind Sciences, National Taiwan UniversityTaipei, Taiwan; ^4^Neurobiology and Cognitive Science Center, National Taiwan UniversityTaipei, Taiwan

**Keywords:** schizophrenia, transmembrane-domain Nrg1 mutant mice, cognitive function, hippocampus, GABAergic interneuron, valproate

## Abstract

Accumulating evidence suggests that neuregulin 1 (NRG1) might be involved in the neurodevelopment, neural plasticity, GABAergic neurotransmission, and pathogenesis of schizophrenia. NRG1 is abundantly expressed in the hippocampus, and emerging studies have begun to reveal the link between NRG1 signaling and cognitive deficits in schizophrenic patients. Because the transmembrane domain of NRG1 is vital for both forward and reverse signaling cascades, new Nrg1-deficient mice that carry a truncation of the transmembrane domain of the Nrg1 gene were characterized and used in this study to test a NRG1 loss-of-function hypothesis for schizophrenia. Both male and female Nrg1 heterozygous mutant mice and their wild-type littermates were used in a series of 4 experiments to characterize the impact of Nrg1 on behavioral phenotypes and to determine the importance of Nrg1 in the regulation of hippocampal neuromorphology and local GABAergic interneurons. First, a comprehensive battery of behavioral tasks indicated that male Nrg1-deficient mice exhibited significant impairments in cognitive functions. Second, pharmacological challenges were conducted and revealed that Nrg1 haploinsufficiency altered GABAergic activity in males. Third, although no genotype-specific neuromorphological alterations were found in the hippocampal CA1 pyramidal neurons, significant reductions in the hippocampal expressions of GAD67 and parvalbumin were revealed in the Nrg1-deficient males. Fourth, chronic treatment with valproate rescued the observed behavioral deficits and hippocampal GAD67 reduction in Nrg1-deficient males. Collectively, these results indicate the potential therapeutic effect of valproate and the importance of Nrg1 in the regulation of cognitive functions and hippocampal GABAergic interneurons, especially in males.

## Introduction

Schizophrenia is a costly and devastating mental disorder that afflicts ~1% of the worldwide population (Insel, 2010). It appears to be a multifactorial disorder with a strong genetic predisposition. Accumulating evidence from human genetic studies suggests that multiple susceptibility genes or loci, including *Neuregulin 1* (*NRG1*) (Schwab and Wildenauer, 2009), might contribute to the pathogenesis of schizophrenia. The association between *NRG1* and schizophrenia was initially revealed in a study of families in Iceland (Stefansson et al., 2002), and the association has been further confirmed in other ethnic groups (Walker et al., 2010). Reduced levels of the expression of NRG1 have also been reported in schizophrenic post-mortem tissues (Bertram et al., 2007; Nicodemus et al., 2009; Parlapani et al., 2010), which indicates that alterations in *NRG1* might contribute to the pathophysiology of schizophrenia.

NRG1, a trophic factor, belongs to the neuregulin family of growth factors, whose effects are mediated via four neuregulin genes (*NRG1-4*) that bind to the ErbB (epidermal growth factor-like receptor) family of tyrosine kinase transmembrane receptors (ErbB1-4) (Harrison and Weinberger, 2005; O'Tuathaigh et al., 2006). As a consequence of multiple promoters and rich alternative splicing, more than 30 different NRG1 isoforms produced from a single *NRG1* gene have been identified to date, and these isoforms have been classified into at least 7 different isoform types (Falls, 2003; Steinthorsdottir et al., 2004; Walss-Bass et al., 2006; Mei and Xiong, 2008). Pro-Neuregulin 1, which contains a transmembrane domain (i.e., the TMc domain, a critical motif for forward and reverse signaling cascades) that forms membrane-anchored precursors, undergoes proteolytic cleavage leading to mature NRG1. NRG1, as a ligand and a receptor for ErbB3 and ErbB4, initiates forward or reverse signaling pathways that have numerous neurotrophic roles (Liu et al., 1998a,b; Bao et al., 2003; Falls, 2003), and NRG1 is abundant in many brain regions, especially in the hippocampus (Law et al., 2004). Numerous roles for NRG1 in CNS development and function have been identified, including synapse formation, neuronal migration, axon guidance, axon myelination, synaptic plasticity, and the regulation of neurotransmitter expression (Harrison and Law, 2006; Mei and Xiong, 2008; Iwakura and Nawa, 2013). The abundant expressions of NRG1 and ErbB and the interactions of these molecules with GABAergic (Yau et al., 2003; Vullhorst et al., 2009; Neddens and Buonanno, 2010), glutamatergic (Hahn et al., 2006; Li et al., 2007), and dopaminergic neurons (Abe et al., 2009; Kato et al., 2011) imply that these molecules have critical roles in the regulation of synaptic plasticity at excitatory and inhibitory synapses that might be involved in the pathogenesis of the cognitive deficits in schizophrenia. Indeed, a novel missense mutation (Val to Leu) in the TMc domain of *NRG1* was reported to be associated with schizophrenia (Walss-Bass et al., 2006), suggesting a potential causal mutations within this gene.

Evidence revealing the link between *Nrg1*/*ErbB4* and cognitive deficits in patients with schizophrenia has begun to accumulate (Hall et al., 2006; Krug et al., 2010). A number of *Nrg1*-related mutant mice have been generated to further elucidate the role of *Nrg1* in the pathogenesis of schizophrenia-related behavioral and cognitive deficits (O'Tuathaigh et al., 2007; Chen et al., 2008; Ehrlichman et al., 2009; Duffy et al., 2010; Wen et al., 2010; Shamir et al., 2012). For example, Nrg1 heterozygous knockout mice with TMc-domain truncation of exon 11 were first reported in 2002 (Stefansson et al., 2002) and this original TMc-*Nrg1*^+/−^ mutant strain has been reported to exhibit behavioral deficits in locomotor activity, explorative behavior, and anxiety-like behaviors (O'Tuathaigh et al., 2006; Boucher et al., 2007). Age-dependent alterations in locomotor activity and exploratory behavior have also been reported in these original TMc-*Nrg1*^+/−^ mutant mice (Karl et al., 2007). Alterations in social and cognitive function have also been reported in these TMc-*Nrg1*^+/−^ mutant mice, such as increased aggression, increased social recognition, decreased prepulse inhibition, and impaired contextual fear conditioning (Stefansson et al., 2002; O'Tuathaigh et al., 2007; Chesworth et al., 2012; Desbonnet et al., 2012). Furthermore, the involvement of *Nrg1* in the modulation of cognitive functions has been further bolstered by *in vitro* electrophysiological studies in the hippocampus of EGF-like domain *Nrg1*^+/−^ mice (Bjarnadottir et al., 2007). A recent study also indicated that ErbB4-null parvalbumin interneuron-restricted mutant mice and EGF-like domain *Nrg1*^+/−^ mice exhibit increased hippocampal LTP (Shamir et al., 2012). These studies suggest the importance of *Nrg1* in the regulation of basic behavioral functions and hippocampal electrophysiology, which might account for the alterations of cognitive functions in these mice. Although some interesting findings were reported, these previous behavioral phenotyping results appear to be somewhat inconsistent across these *Nrg1*-related mutant mice or even across variants of some lines of *Nrg1* mutant mice from one study to another. It is also difficult to make direct comparisons between different lines of mutant mice because different gene targeting strategies were used in different studies and because the expression levels of Nrg1 protein in these haploinsufficient mice are not available due to the lack of antibodies that are capable of recognizing the different isoforms. Besides, in the past decade, most of Nrg1-related mouse studies were conducted in male mice and only a few studies used either female mice or both male and female mice. Interestingly, sex-specific effects were reported. For example, using both male and female TMc-*Nrg1*^+/−^ mutant mice, it was previously found that only male mutant mice had impaired performance in Barnes maze (O'Tuathaigh et al., 2007) and only female mutant mice had reduced grooming behavior (O'Tuathaigh et al., 2006). It is of interest to further compare sex-specific effect on different behavioral tasks in Nrg1-related mutant mice.

To further characterize the effects of *Nrg1* haploinsufficiency and to validate the impact of the complete truncation of the *Nrg1* TMc-domain on cognitive functioning and its behavioral consequences in both males and females, a novel line of TMc-domain *Nrg1*^+/−^ mutant mice with a truncation from exon 9 was obtained and used in this study. A set of four experiments was designed. Because sex (or sex hormones) might have differential effects on NRG/ErbB via glial-neuronal signaling in schizophrenic patients (Lacroix-Fralish et al., 2006; Wong and Weickert, 2009) and because sex-specific effects of Nrg1 on behavioral phenotypes have not been well characterized in mice, both male and female mice were used in Experiments 1–3. In Experiment 1, a comprehensive battery of cognitive-related tasks (Experiment 1A) and basic behavioral tasks (Experiment 1B) was applied to evaluate the behavioral phenotype of the novel TMc-*Nrg1*^+/−^ mutant mice. Experiments 2 and 3 were conducted to facilitate the interpretation of the behavioral deficits observed in Experiment 1. Accordingly, in Experiment 2, MK-801 (an NMDA receptor antagonist), methamphetamine (a potent psychostimulant that increases the amount of extracellular dopamine), and pentylenetetrazol (PTZ, a GABA_A_ receptor antagonist) were used to induce behavioral alterations and to reveal which neurotransmitters were more vulnerable in these novel TMc-*Nrg1*^+/−^ mutant mice. Neuromorphological and neurochemical alterations in the hippocampi of these mutant mice were further examined in Experiments 3A and 3B, respectively. Based on the findings from the first 3 experiments, an effective and useful procedure of valproate (a *GABA* transaminase inhibitor) treatment (Guidotti et al., 2009) was used to evaluate whether rescue of the observed cognitive deficits in these novel TMc-*Nrg1*^+/−^ mice was possible.

## Materials and methods

### Animals

All *neuregulin 1* heterozygous mutant mice (*Nrg1*^+/−^) and wild-type (WT) littermate mice used in this study were generated from *Nrg1*^+/−^ breeding pairs with C57BL/6J genetic backgrounds (*n* > 10). Only *Nrg1*^+/−^ mice were used because *Nrg1* homozygous embryos die due to cardiac defects around E10.5–E11.5 (Gassmann et al., 1995; Meyer and Birchmeier, 1995; Kramer et al., 1996). *Nrg1*^+/−^ mice were healthy, and basic physical examination revealed no observable physical damage. Mice were weaned at 4 weeks and separated by sex. Littermates of the same sex were caged separately and maintained in groups of 3–5 mice per cage. For behavioral testing, each mouse was housed individually 1 week before behavioral testing. All mice were housed, with food and water provided *ad libitum*, in individually ventilated polysulfone cages (Alternative design Inc., U.S.A.) within the animal rooms of the Psychology Department of National Taiwan University. All animals used in this study were adult mice (at least 2 months of age). They were handled and weighed daily for at least 1 week prior to each experiment. All behavioral and pharmacological experiments were conducted in the dark phase. Minimal numbers of mice were used to meet the 3R reduction principle of animal use. All animal procedures were performed according to protocols approved by the Animal Care and Use Committee of National Taiwan University. Adequate measures were taken to minimize potential pain and discomfort that the mice used in this study may have experienced.

### Obtainment and validation of the novel TMc-*Nrg1*^+/−^ mice

**Figure 1:**
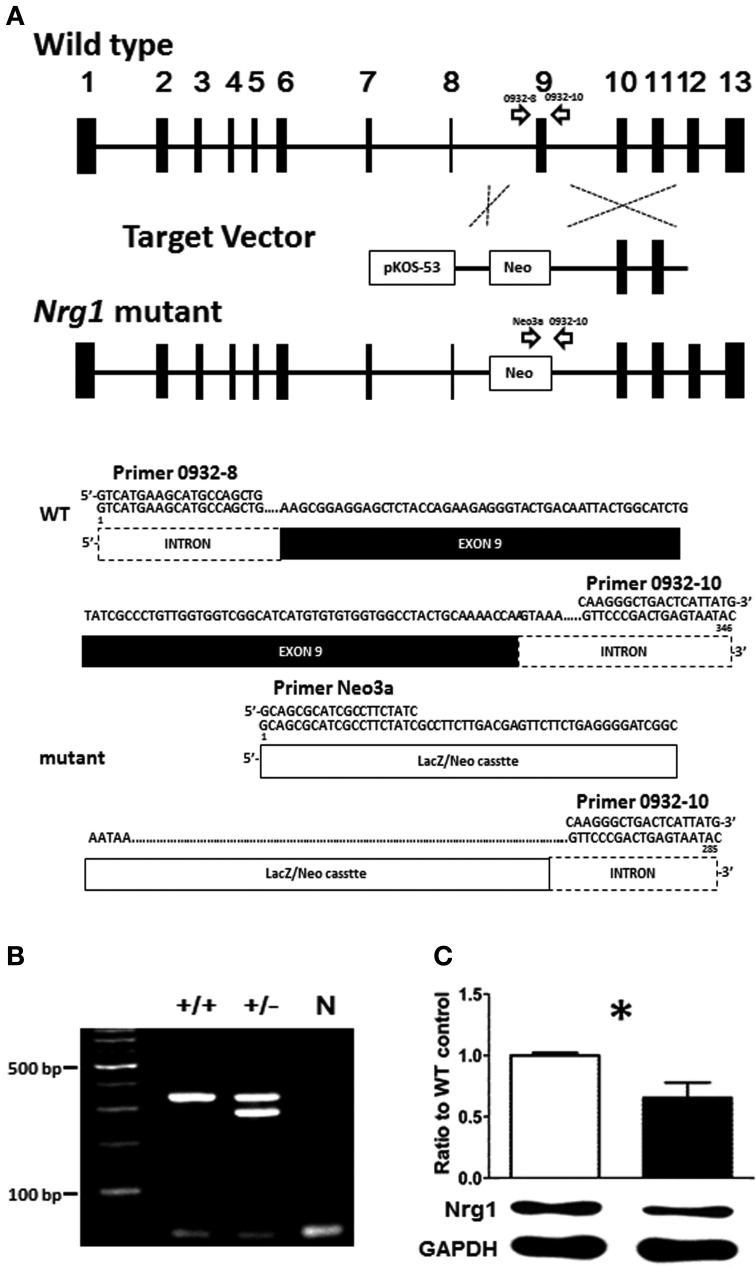
**(A)** Schematic diagram of the targeting strategy for the novel TMc-*Nrg1*^+/−^ mutant mice. Upper panel, wild type (WT) *Nrg1* locus including 13 exons, shown as black boxes. The target vector, pKOS-53 with a LacZ/Neo cassette, was targeted to exon 9 of *Nrg1* by homologous recombination. Lower panel, primer 0932-8, primer 0932-10, and primer Neo3a were used to distinguish WT alleles (346 bps) from mutant alleles (258 bps). **(B)** Mouse-tail DNA was submitted to PCR analysis. N: negative control. **(C)** The expression level of Nrg1 proteins in the brain of both WT and *Nrg1*^+/−^ mice. ^*^P < 0.05 between two genotypes.

Given the fact that the TMc domain of NRG1 is a critical motif for NRG1 forward and reverse signaling cascades and that reduced levels of the expression of NRG1 have been reported in some schizophrenic post-mortem tissues, the validation of this novel TMc-*Nrg1* mutant mouse strain is important and this mouse strain is beneficial to study the effect of TMc domain of *Nrg1* in the pathogenesis of schizophrenia-related symptoms. In complementary to other Nrg1-related mouse strains, this novel strain of mice is also helpful to elucidate the functional consequences of TMc domain ablation and the impact of *Nrg1* TMc-domain complete truncation on behavioral functions. The novel TMc-*Nrg1* mutant mice were originally generated by Lexicon Genetics (NIH-0932, LEXKO-1007) using pKOS-53 with LacZ/Neo cassettes. Using homologous recombination, exon 9 of *Nrg1*, where it was translated into the TMc domain, were targeted and truncated by the LacZ/Neo cassettes. Further information about this mouse has been described previously (http://www.informatics.jax.org/external/ko/lexicon/4608.html). A schematic diagram of the targeting strategy is depicted in Figure 1A. The entire sequence encoding the TMc is encoded by a single exon so that the truncated exon can be referred as the TMc exon. Likely due to differences in exon annotations, the targeted TMc exon was previously referred to as exon 11 in the original TMc-*Nrg1* mutant strain (Stefansson et al., 2002) and it was referred to as exon 9 in our novel TMc-*Nrg1* mutant strain here. The expected sizes of the PCR products for the novel TMc-Nrg1 mice are indicated in Figure 1A as well. Genomic DNA isolated from the tails of the mice of the indicated genotypes was submitted to PCR, and WT and recombinant alleles were distinguished with agarose gel electrophoresis (Figure 1B). The following primers were used to confirm the genotypes of the mice: 0932-8, 5′-GTCATGAAGCATGCCAGCTG-3′; 0932-10, 5′-GTATTACTCAGTCGGGAAC-3′; and Neo3a, 5′-GCAGCGCATCGCCTTCTATC-3′ (Figure 1). The TMc domain of *Nrg1* was truncated by the target vector, which resulted in the lack of a C-terminal on Nrg1 and a resistance to proteolytic release. The deletion included the sequence VLTITGICIALLVVGIMCVVAY, which has been described previously as the TMc domain of *Nrg1* (Walss-Bass et al., 2006). The truncated region starts with a hydrophilic part and the majority in this region appears to be hydrophobic (average hydrophobicity = 1.233; >1 means this region is hydrophobic) based on analyses with SOSUI (i.e., a classification and secondary structure prediction system for membrane proteins) (Hirokawa et al., 1998). The result of hydrophobicity analysis predicts that this region is in the very 5′ region of the TMc-domain of Nrg1 and it is translated as a hydrophobic protein structure because most of the amino acids in the TMc-domain are hydrophobic. The expression of Nrg1 protein in the cerebral cortex of the novel TMc-*Nrg1*^+/−^ mutant mice was further validated using Western blot with a *Nrg1* C-terminal antibody (Neuregulin-1α/β1/2, 1:500, sc348, Santa Cruz Biotechnology). As indicated in Figure 1C, compared to WT littermate controls, a significant (~37%) reduction of Nrg1 protein (~51 kDa) was found in the brains of the novel TMc-*Nrg1*^+/−^ mice [WT: 1 ± 0.013; *Nrg1*^+/−^: 0.628 ± 0.110; *t*_(4)_ = 3.373; *p* = 0.027].

### Experiment 1: examination of the cognitive and basic behavioral functioning of the novel TMc-*Nrg1*^+/−^ mutant mice

Two cohorts of male and female adult TMc-*Nrg1*^+/−^ mice and their WT littermates were used in this experiment. Two series of experiments were conducted to examine the effects of Nrg1 haploinsufficiency on the cognitive functions (Experiment 1A) and the basic behaviors (Experiment 1B) in these mice. Cognitive function-related behavioral tasks used in this experiment included the object recognition task, contextual and cued fear conditioning, the Morris water maze task, prepulse inhibition, and social preference and recognition task. The object recognition task, contextual fear conditioning, Morris water maze, and social recognition task are considered to be related to hippocampus-dependent function. Cued fear conditioning is considered to rely heavily upon the amygdala. Prepulse inhibition was performed to measure sensorimotor gating function in these mice. Basic behaviors (Experiment 1B) were evaluated with a battery of behavioral tasks that consisted of an open-field locomotor assay, a hole board task, an elevated plus maze task, a sucrose preference task, and a hot plate task. Because it has been reported that some basic behaviors might be age-dependent in the original TMc-*Nrg1* mutant mice (Karl et al., 2007), both novel TMc-*Nrg1*^+/−^ and WT mice were tested twice at ages close to 2 and 6 months for basic behavioral phenotyping and clarification of age-dependent effects. Two cohorts of mice were used. The first cohort of mice (*n* = 12 each) was sequentially tested with the novel object recognition task on post-natal days 63–65 (PND 63–65), with the fear conditioning task on PND 66–70 and with the Morris water maze on PND 90–110. The other cohort of mice (male = 12; female = 10) was tested with the open field task on PND 63 and PND 189, with the hole board task on PND 65 and PND 191, the elevated plus maze task on PND 67 and PND 193, the social preference and recognition task on PND 71, the sucrose preference task on PND 72–75 and PND 199–202, and the prepulse inhibition task on PND 78. The details of the behavioral tasks are briefly described in the Supplementary Materials. These behavioral tasks have been evaluated with varying degrees of test validities for assessing schizophrenia-relevant behavioral deficits in mice (Brooks and Dunnett, 2009; Lai et al., 2014).

### Experiment 2: evaluation of drug-induced behavioral alterations in the novel TMc-*Nrg1*^+/−^ mice

Based on three major neurotransmitter-based hypotheses of schizophrenia (i.e., the dopamine, glutamate, and GABA hypotheses), three related drugs were selected to investigate which neurotransmitter system was most vulnerable to the alterations of behavioral performance observed in our TMc-*Nrg1*^+/−^ mutant mice. Three cohorts of male and female TMc-*Nrg1*^+/−^ and WT adult (2 months of age; *n* = 12 each) mice were used in this experiment. Mice from Experiments 2A–C received an acute administration of MK-801 (an NMDA receptor antagonist, 0.25 mg/kg, i.p.), methamphetamine (a potent psychostimulant, 2 mg/kg, i.p.), or pentylenetetrazol (PTZ, a GABA_A_ receptor antagonist, 60 mg/kg, s.c.). The volume of administration was 5 ml/kg and the vehicle used in this study was saline (0.9%). The subsequent behaviors of these mice in a polyvinylchloride chamber (48 × 24 × 25 cm) were recorded using an EthoVision video tracking system or ETHOM software (Shih and Mok, 2000). On the day of the measurements, the total distances traveled (cm) by both the male and female mice in the first cohort were recorded in the chamber for 60 min after the administration of saline (0.9%, 5 ml/kg) as a baseline. Then, locomotor activity was recorded for two 60-min periods after the injection of MK-801. For the second cohort of male and female mice, total distances traveled (cm) were also recorded for a 60-min baseline period following the administration of saline and for two 60-min periods following methamphetamine injection. The doses of MK-801 and methamphetamine were chosen to avoid stereotypic behaviors in the open field as described previously (Wu et al., 2005; Van Den Buuse et al., 2009; Chen et al., 2012, 2014). The third cohort of male and female mice received an injection of PTZ to induce a seizure response, and their behaviors were videotaped and recorded in the chamber for 1 h. The dose of PTZ was chosen based on previous dose studies of C57BL/6 mice (Itoh and Watanabe, 2009). The severities of the seizure responses were scored blindly using the following previously described scale: 0, no response; 1, hypoactivity; 2, partial clonus; 3, generalized clonus; and 4, tonic-clonic (maximal) seizure (Ferraro et al., 1999, 2010).

### Experiment 3: examination of the neuromorphological and neurochemical alterations in the hippocampi of the novel TMc-*Nrg1*^+/−^ mice

Based on the behavioral deficits observed in Experiment 1A, Experiment 3 concentrated on examination of the neuromorphological alterations of the hippocampi of mutant and WT mice. Based on the findings regarding the drug-induced behavioral alterations of the TMc-*Nrg1*^+/−^ mutant mice in Experiment 2C, we further examined the expression of GABAergic markers in the hippocampi of these mutant mice. Both male and female TMc-*Nrg1*^+/−^ and WT adult (2–4 months of age) mice were used in this experiment. Neuromorphological analyses were first conducted to evaluate whether the haploinsufficieny of Nrg1 resulted in neuromorphological alterations of the pyramidal neurons of the CA1 region of the hippocampus (Experiment 3A). Hippocampal samples from both mutant and WT mice were harvested after behavioral testing to examine protein expression using Western blot (Experiment 3B).

#### Experiment 3A—neuromorphological analysis of hippocampal CA1 pyramidal neurons

As a follow up to the observations of hippocampal-dependent impairments in the novel TMc-*Nrg1*^+/−^ mutant mice, hippocampi were examined for any neuromorphological alterations in the mutant mice that could partially account for the behavioral deficits. Because pyramidal neurons of the CA1 region of the hippocampus are critical for the afferent and efferent connections of the hippocampus, the C57BL6-*Tg* (GFPm) driven by the Thy1 promoter specific transgenic mouse line was selected and used for analyses of the neuromorphologies of GFP (green fluorescent protein)-labeled CA1 pyramidal neurons of the hippocampus (Feng et al., 2000). The expression patterns of GFP-labeled pyramidal neurons in Thy1-C57BL6-*Tg*(GFPm) transgenic mouse line was reported previously (Feng et al., 2000) and this mouse line has been successfully used to analyze neuromorphological alterations of pyramidal neurons in mutant mice (Lai et al., 2006; Chen and Lai, 2011). Based on the hippocampus-dependent behavioral deficits observed in the novel TMc-*Nrg1*^+/−^mutant mice, additional male and female subjects generated from our TMc-*Nrg1*^+/−^ breeding pairs with C57BL6-*Tg* (GFPm) backgrounds were used in this experiment. Adult mice were anesthetized and transcardially perfused with PBS followed by 4% paraformaldehyde in PBS. Fixed brains were sectioned coronally using a vibratome. A series of 150-μm coronal sections were collected and mounted on slides for scanning. Confocal microscopy stack images of GFP-labeled neurons were obtained at intervals of 0.4 μm using 20×, 40×-oil, and 63×-oil objectives from a Leica TCS SP5 confocal microsystem (Leica, Taipei, Taiwan). The Neurolucida software (MicroBrightField Incorporated, Williston, VT, USA) was used to trace and reconstruct the neurons in 3 dimensions. GFP labeling was nearly exclusively restricted to the cell bodies and dendritic trees of CA1 pyramidal neurons of both mutant mice and their littermate controls. Using Neurolucida software, morphometric analyses of the GFP-labeled pyramidal neurons (between Bregma −1.46 and −2.80 mm) of the TMc-*Nrg1*^+/−^ mice and WT littermate controls were performed (total *n* ≧ 4 each group) to reveal the neuromorphological differences between genotypes. The following 14 morphological variables were chosen based on previous studies (Lai et al., 2006; Chen and Lai, 2011): (1) soma size (cell soma sizes were obtained by outlining cell somas and automatically calculating the pixel areas in μm^2^); (2) the distance to apical bifurcation (base of the apical tuft) measured from the cell body to the major branch point of the apical dendrite; (3) the number of branches on the apical branches; (4) the number of apical tips; (5) the total length of the apical tuft, which was taken as the sum of the lengths of the apical stem and the branches that formed the tuft; (6) the apical dendritic field area (ADFA), which measures the area of the dendritic field of a neuron and is calculated as the area enclosed by a polygon that joins the most distal points of the dendritic processes (convex area); (7) the branch angles of the primary apical dendrites (from the distal end of apical bifurcation); (8) the number of primary basal dendrites (not including apical dendrites and axons); (9) the total length of the primary basal dendrites; (10) the number of branches from the basal branches; (11) the number of basal tips; (12) the total length of the basal dendrites; (13) the basal dendritic field area (BDFA), which measures the area of the dendritic field of a neuron and is calculated as the area enclosed by a polygon that joins the most distal points of the dendritic processes (convex area); and (14) Sholl analysis of basal dendritic complexity.

#### Experiment 3B—examination of the expression of GABAergic markers in the hippocampi of the novel TMc-Nrg1^+/−^ mice

After finishing behavioral testing for cognitive functions in Experiment 1A, some mice from Experiment 1A were randomly selected and used to examine the expression of hippocampal GABAergic markers in this experiment. Hippocampi from both WT and TMc-*Nrg1*^+/−^ male (*n* = 7 each) and female (*n* = 6 each) adult mice (3–4 months of age) were quickly dissected, frozen in liquid nitrogen, and stored at −80°C. Tissue samples were homogenized in lysis buffer containing 25 mM Tris (pH 8.0), 125 mM NaCl, Protease Inhibitor Cocktail tablets (Roche, Taipei, Taiwan) and Phosphatase Inhibitor Cocktail 1 (P2850, Sigma-Aldrich, St. Louis, MO, USA) and then centrifuged at 12,500 rpm (14324 g) at 4°C for 15 min. The supernatant was then collected. The protein concentrations of the supernatants were measured with the Bradford protein assay (Bio-Rad Laboratories, Tokyo, Japan) and spectrometry at 620 nm. The same amount of protein was separated by 4–10% sodium dodecyl sulfate/polyacrylamide gel electrophoresis (SDS/PAGE) and transferred onto a nitrocellulose membrane (Millipore, Billerica, MA, USA). Ponceau S staining of the membrane was used to ensure successful transfer. Subsequently, the membranes were washed in Tris-buffered saline containing 0.1% Tween-20 (TBST) and blocked in 5% (w/v) skim milk for 1 h at 25°C. The membrane was then incubated in a 5% (w/v) skim milk solution with the appropriate primary antibody overnight at 4°C. The blots were probed with the following antibodies: GAD67 (1:5000, MAB5406, Millipore), calretinin (1:2000, AB5054, Millipore), parvalbumin (1:2000, P3088, Sigma-Aldrich), and GAPDH (1:5000, #2118, Cell Signaling Technology, Inc., Danvers, MA, USA). Immune complexes were visualized using the appropriate peroxidase-conjugated secondary antibodies (Cell Signaling Technology). Bound antibody was detected using an enhanced chemiluminescence (ECL) kit (Millipore), and densitometric analysis was performed using Image J (a Java-based image processing program developed at the National Institutes of Health).

### Experiment 4: evaluation of the effect of valproate on the rescue of cognitive deficits in the novel TMc-*Nrg1*^+/−^ male mice

Valproate, a *GABAergic* anti-epileptic drug and a potential pharmacoepigenetic agent, has been reported to facilitate GABAergic-promoter demethylation, to inhibit histone deacetylase via chromatin remodeling, and to enhance the expression of GABA-related genes (Guidotti et al., 2009, 2011). To evaluate rescue of the observed behavioral deficits that resulted from GABAergic alternation in young adult (3–4 months of age) TMc-*Nrg1*^+/−^ male mice, valproate (VPA, 1.5 mmol/kg, s.c.) or vehicle (0.9% saline, 10 ml/kg) was injected into male TMc-*Nrg1*^+/−^and WT mice twice daily for 17 days (WT/saline: *n* = 6; WT/VPA: *n* = 7; *Nrg1*^+/−^/saline: *n* = 8; *Nrg1*^+/−^/VPA: *n* = 8). Based on the findings from Experiment 1A, two behavioral tasks were selected to evaluate the drug's efficacy in rescuing hippocampal function in the mutant mice. The object recognition task was conducted on day 16. Fear conditioning training was conducted on day 16, and contextual/cued tests of fear conditioning were conducted on day 17. Prior to behavioral testing, each subject was left untreated for at least 12 h to exclude any acute effects of daily valproate injection. The behavioral procedures were nearly identical to those described for Experiment 1A, with the exception that the cued fear conditioning tests were conducted 1 h, rather than 1 day, after contextual fear conditioning to reduce the number of testing days. One day after last behavioral testing (on day 18), whole hippocampi from these mice were dissected and processed for Western blotting using GAD67 antibody as described previously in Experiment 3B.

### Statistical analysis

All data are presented as the mean ± standard error of the mean (SEM). Statistical analyses were performed using SPSS 13.0 (SPSS Inc., Chicago, IL, USA), and the data were analyzed with One- or Two-Way ANOVAs or two-sample Student's *t*-tests, where appropriate. *Post-hoc* analyses were performed using Fisher's LSD tests when the *F*-values revealed significant differences. A priori *t*-tests (with Bonferroni adjustments when needed) were conducted to compare genotype-dependent differences and to examine specific hypotheses. *P* values of < 0.05 were considered statistically significant.

## Results

### Experiment 1: the novel TMc-*Nrg1*^+/−^ mice have normal basic behavioral functioning and impairments in hippocampal- dependent cognitive functions

#### Experiment 1A—examination of cognitive-related function

**Figure 2:**
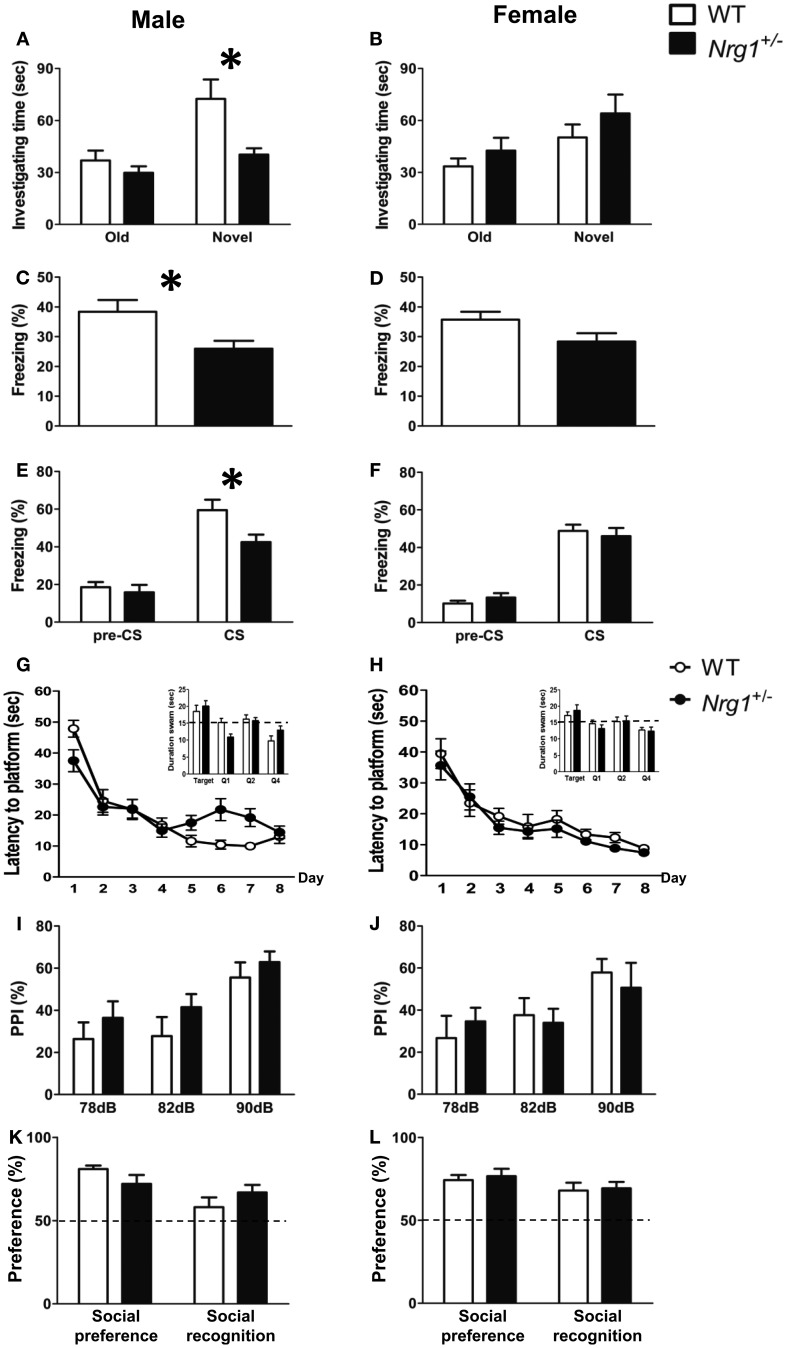
**The cognitive functions (mean + s.e.m.) of both male and female TMc-*Nrg1*^+/−^ mutant mice (black bars) and their wild-type littermate controls (WT, white bars) were evaluated in the Experiment 1 of this study. (A,B)** In the object recognition task, a reduction in investigation time (s) of the novel object was observed in male, but not female, TMc-*Nrg1*^+/−^ mice. **(C,D)** In the contextual fear conditioning task, a reduction in freezing time percentage was observed in male, but not female, TMc-*Nrg1*^+/−^ mice. **(E,F)** In the cued fear conditioning task, a reduction in freezing time percentage (%) was found in male, but not female, TMc-*Nrg1*^+/−^ mice. In contrast, no significant genotype-dependent differences were found in the Morris water maze, prepulse inhibition task, or the social task in either males **(G,I,K)** or females **(H,J,L)**, respectively. Dash lines indicate chance level. ^*^P < 0.05 between two genotypes.

A total of five behavioral tasks were conducted in this experiment. In the object recognition task, a Two-Way ANOVA revealed a significant interaction of genotype and sex [*F*_(1, 29)_ = 7.222, *p* = 0.012] on the time spent investigating a novel object. As depicted in Figures 2A,B, further statistical analyses reveal a significant simple main effect of genotype for the males (*p* = 0.011) but not the females (*p* = 0.309). Because we intended to examine the sex-specific effects of Nrg1 in mice and also because we found a sex difference, the following data analyses were carried out independently for each sex. During fear conditioning, the freezing responses of male TMc-*Nrg1*^+/−^ mice in both contextual [*t*_(22)_ = −2.622, *p* = 0.016; Figure 2C] and cued [*t*_(22)_ = −2.487, *p* = 0.021; Figure 2E] fear conditioning were significantly reduced compared to WT controls. In contrast, no significant differences due to genotype were found for the female mice (both *p* > 0.05; Figures 2D,F). In the Morris water maze, no significant differences due to genotype were found for either the male or female mice during the learning phase (both *p* > 0.05) or the probe phase (both *p* > 0.05), as depicted in Figures 2G,H. Regarding sensorimotor gating functions, no genotype-dependent differences were present in either the males (*p* > 0.05; Figure 2I) or the females (*p* > 0.05; Figure 2J). No genotype-dependent differences were found in their startle response and startle habituation as well. In the social preference and social recognition tasks, no significant genotype-dependent differences were found in either the males (*p* > 0.05; Figure 2K) or the females (*p* > 0.05; Figure 2L).

#### Experiment 1B—basic behavioral phenotyping

The basic functioning of the novel TMc-*Nrg1*^+/−^ mice was further examined using a battery of behavioral tasks. As illustrated in Table 1, relative to the WT mice, both 2-month-old male and female TMc-*Nrg1*^+/−^ mice displayed normal behavioral profiles across a series of basic behavioral tasks that included the open field, hole board, elevated plus maze, 2% sucrose preference, and hot plate tasks (all *ps* > 0.05). Because age-dependent effects have been previously reported for some *Nrg1* mutant mice, these mice were examined again with the same battery of behavioral tasks at 6 months of age. Again, no genotype-dependent differences were found in either the males or females (Table 1).

**Table 1:** **A summary of the basic behavioral phenotyping and statistical analyses (mean ± s.e.m) in the novel TMc-*Nrg1*^+/−^ (*Nrg1*^+/−^) mutant mice and their wild-type (WT) littermates**.

**Behavioral task**	**2-month-old**				**6-month-old**			
	**Male**		**Female**		**Male**		**Female**	
	**WT**	***Nrg1*^+/−^**	**WT**	***Nrg1*^+/−^**	**WT**	***Nrg1*^+/−^**	**WT**	***Nrg1*^+/−^**
Open field	PND 63		PND 63		PND 189		PND 189	
**Aversive ratio (central zone/total duration)**	0.10 ± 0.12	0.27 ± 0.08	0.11 ± 0.01	0.18 ± 0.08	0.15 ± 0.02	0.21 ± 0.06	0.13 ± 0.01	0.23 ± 0.07
**Travel distance (cm)**	19350.85 ± 759.67	20391.52 ± 1107.93	17909.69 ± 1688.31	19105.10 ± 1904.06	21729.07 ± 1115.63	22962.49 ± 1272.84	19410.62 ± 1368.97	22920.24 ± 1834.40
**Travel distance habituation**								
First 10-min (cm)	4774.00 ± 268.28	4931.53 ± 226.45	4470.28 ± 390.28	4844.71 ± 390.78	4560.65 ± 250.31	4915.65 ± 254.11	4261.78 ± 311.75	5216.76 ± 419.29
Second 10-min (cm)	3509.14 ± 219.53	3786.85 ± 283.86	3104.64 ± 374.80	3071.39 ± 440.58	3617.72 ± 251.13	4073.96 ± 191.76	3158.06 ± 267.98	3853.18 ± 335.61
Third 10-min (cm)	3344.77 ± 243.33	3263.94 ± 358.69	2732.59 ± 307.71	2905.19 ± 432.88	3679.20 ± 178.48	3679.68 ± 255.05	3239.92 ± 260.67	3596.45 ± 290.17
Fourth 10-min (cm)	2847.36 ± 159.58	2939.80 ± 197.43	2636.30 ± 272.01	3549.86 ± 547.15	3508.67 ± 253.71	3549.91 ± 254.77	3039.12 ± 257.56	3680.28 ± 290.03
Fifth 10-min (cm)	2564.62 ± 186.07	2946.20 ± 237.96	2671.88 ± 286.70	2419.68 ± 299.39	3417.01 ± 176.23	3408.28 ± 293.49	2905.29 ± 244.49	3376.65 ± 326.67
Sixth 10-min (cm)	2310.97 ± 147.14	2523.20 ± 170.86	2294.01 ± 361.53	2314.27 ± 414.70	2945.82 ± 181.92	3335.02 ± 233.27	2806.45 ± 260.11	3196.92 ± 314.25
Hole board	PND 65		PND 65		PND 191		PND 191	
**Number of head dipping (#)**	201.67 ± 25.95	189.17 ± 35.00	233.80 ± 52.52	238.90 ± 38.37	124.33 ± 20.19	140.67 ± 21.37	153.40 ± 18.11	180.40 ± 18.01
**Travel distance (cm)**	6508.39 ± 289.29	6866.35 ± 243.81	6343.31 ± 353.52	7127.08 ± 337.22	6457.39 ± 239.37	6487.12 ± 364.16	6090.96 ± 236.28	7112.74 ± 318.51
**Head dipping habituation**								
First 5-min (#)	40.75 ± 5.09	36.50 ± 5.58	51.80 ± 10.50	51.50 ± 6.37	21.75 ± 3.29	28.58 ± 4.78	30.20 ± 4.44	42.00 ± 5.63
Second 5-min (#)	39.67 ± 5.38	36.25 ± 6.42	44.90 ± 10.39	48.30 ± 8.27	24.17 ± 4.92	27.92 ± 5.76	30.60 ± 3.79	32.30 ± 4.48
Third 5-min (#)	36.08 ± 5.75	30.00 ± 5.85	37.90 ± 8.48	40.80 ± 6.68	20.42 ± 3.43	22.00 ± 3.05	24.70 ± 3.93	30.10 ± 3.39
Fourth 5-min (#)	30.75 ± 4.76	31.83 ± 7.01	38.80 ± 13.00	37.60 ± 8.48	19.67 ± 5.67	22.00 ± 3.06	24.30 ± 4.73	26.00 ± 3.35
Fifth 5-min (#)	29.00 ± 4.78	28.17 ± 6.30	32.70 ± 7.69	32.80 ± 7.39	20.58 ± 3.74	23.00 ± 4.69	24.10 ± 4.96	26.90 ± 4.25
Sixth 5-min (#)	25.42 ± 4.18	26.42 ± 5.70	27.70 ± 5.01	27.90 ± 4.54	17.75 ± 3.14	17.17 ± 3.73	19.50 ± 3.62	23.10 ± 2.77
**Travel distance habituation**								
First 5-min (cm)	1269.49 ± 40.78	1357.08 ± 65.25	1289.60 ± 69.55	1392.35 ± 61.02	1330.91 ± 55.53	1328.89 ± 88.20	1282.37 ± 33.57	1381.22 ± 83.26
Second 5-min (cm)	1132.73 ± 44.03	1215.48 ± 62.73	1130.86 ± 68.89	1246.50 ± 49.04	1119.68 ± 43.96	1108.36 ± 81.33	1061.13 ± 37.29	1184.67 ± 55.76
Third 5-min (cm)	1070.93 ± 40.15	1097.51 ± 44.89	1055.56 ± 64.15	1178.20 ± 59.55	1044.60 ± 44.07	1082.08 ± 59.39	943.84 ± 52.58	1184.59 ± 73.09
Fourth 5-min (cm)	1093.80 ± 52.36	1116.40 ± 41.85	977.99 ± 61.45	1115.24 ± 82.81	1045.09 ± 57.52	1030.64 ± 66.32	996.34 ± 42.75	1174.38 ± 63.02
Fifth 5-min (cm)	995.75 ± 68.49	1075.98 ± 39.52	998.38 ± 66.39	1163.19 ± 103.82	985.70 ± 35.07	963.88 ± 45.66	920.71 ± 45.97	1065.95 ± 76.68
Sixth 5-min (cm)	945.69 ± 75.63	1003.89 ± 43.86	890.92 ± 90.25	1031.60 ± 61.97	931.41 ± 57.90	973.27 ± 75.32	886.57 ± 69.46	1121.93 ± 62.33
Elevated plus maze	PND 67		PND 67		PND 193		PND 193	
**Aversive ratio (open arm/total duration)**	0.20 ± 0.03	0.29 ± 0.05	0.23 ± 0.04	0.29 ± 0.03	0.18 ± 0.06	0.21 ± 0.05	0.17 ± 0.03	0.25 ± 0.05
**Number of head dipping (#)**	11.92 ± 2.05	15.83 ± 1.99	15.40 ± 2.54	15.80 ± 1.45	10.17 ± 2.50	9.58 ± 1.91	10.20 ± 1.30	12.10 ± 2.16
**Number of rearing (#)**	10.42 ± 1.10	13.42 ± 2.05	13.00 ± 2.16	12.10 ± 1.91	11.58 ± 1.76	14.00 ± 1.86	15.10 ± 2.05	13.90 ± 2.34
Sucrose preference	PND 72–75		PND 72–75		PND 199–202		PND 199–202	
**2% sucrose preference (%)**	78.78 ± 2.10	80.32 ± 1.80	82.21 ± 1.74	81.59 ± 2.14	81.07 ± 1.94	80.35 ± 3.56	83.96 ± 3.28	78.03 ± 3.31
**2% sucrose preference habituation**								
Day 1 sucrose preference (%)	75.68 ± 2.19	81.66 ± 1.97	81.12 ± 1.97	76.87 ± 5.58	81.98 ± 2.23	74.41 ± 5.85	78.49 ± 5.65	71.36 ± 8.27
Day 2 sucrose preference (%)	77.42 ± 4.95	82.54 ± 1.24	80.60 ± 4.72	83.77 ± 2.10	80.40 ± 3.71	79.43 ± 5.77	83.95 ± 7.26	78.94 ± 4.65
Day 3 sucrose preference (%)	83.25 ± 1.27	76.77 ± 5.77	84.83 ± 3.02	84.14 ± 1.41	80.84 ± 4.54	87.20 ± 2.35	87.73 ± 2.12	83.81 ± 3.59

A series of four basic behavioral tests (left column, from top to bottom), including the open-field task, the hole board task, the elevated plus maze, and the sucrose preference task, were conducted repeatedly at ages close to 2 and 6 months in these mice. There were no significant differences between genotypes in either sex at any age. PND, post-natal day.

### Experiment 2: evaluation of drug-induced behavioral alterations in the novel TMc-*Nrg1*^+/−^ mice

**Figure 3:**
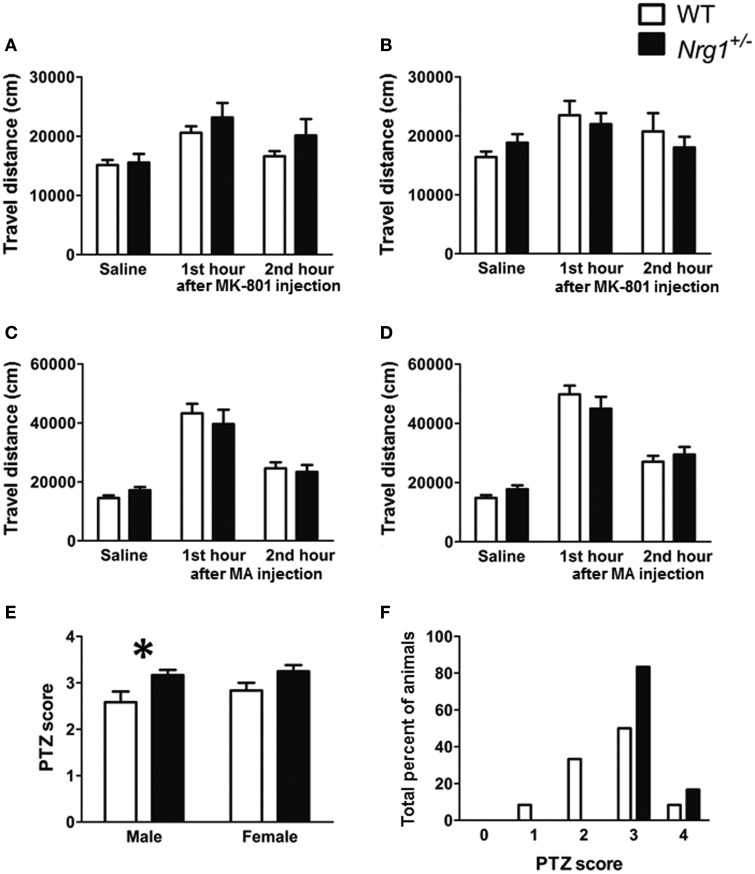
**Drug-induced behavioral alterations (mean + s.e.m.) in the novel TMc-*Nrg1*^+/−^ mice (black bars) and their wild-type controls (WT, white bars) (*n* = 12 each group) of this study. (A,B)** Total travel distances for the two groups of male and female mice during the 1-h saline baseline and the 1st and 2nd 1-h periods after the injection of MK-801 (0.25 mg/kg, i.p.). **(C,D)** Total travel distances (mean + s.e.m.) for the two groups of male and female mice during the 1-h saline baseline and during the 1st and 2nd 1-h periods after the injection of methamphetamine (MA, 2 mg/kg i.p.). **(E)** PTZ (60 mg/kg, s.c.)-induced seizure severity scores [from 0 (no response) to 4 (maximal seizure)] for both male and female mice. **(F)** Distributions of PTZ-induced seizure severity scores for male mice (%). ^*^P < 0.05 between two genotypes.

Three cohorts of male and female TMc-*Nrg1*^+/−^ and WT adult mice were used to evaluate MK-801-, methamphetamine-, and PTZ-induced behavioral alterations in Experiments 2A–C, respectively. In Experiment 2A, a significant MK-801 treatment effect was found in both males [*F*_(2, 44)_ = 15.505, *p* < 0.001] and females [*F*_(2, 44)_ = 5.188, *p* = 0.009]. But no significant genotype-dependent differences were found in either the 60-min baseline activity or MK-801-induced hyperlocomotion in either the male (Figure 3A) or female mice (Figure 3B). Similarly, in Experiment 2B, a significant methamphetamine treatment effect was found in both males [*F*_(2, 44)_ = 59.680, *p* < 0.001] and females [*F*_(2, 44)_ = 126.353, *p* < 0.001]. But no significant genotype-dependent differences were found in either the 60-min baseline activity or methamphetamine-induced hyperlocomotion in either the male (Figure 3C) or female mice (Figure 3D). In contrast to the results of Experiments 2A and 2B, the severities of PTZ-induced seizures were significantly greater in male TMc-*Nrg1*^+/−^ mice [*t*_(22)_ = −2.288, *p* = 0.032; Figure 3E] but not in female TMc-*Nrg1*^+/−^ mutant mice compared their respective WT controls. This genotype-dependent deficit of the males is further illustrated in Figure 3F, which shows the distributions of PTZ-induced seizure scores (Figure 3F).

### Experiment 3: examination of neuromorphological and neurochemical alterations in the hippocampi of the novel TMc-*Nrg1*^+/−^ mice

#### Experiment 3A—neuromorphological analysis

Based on the cognitive deficits observed in Experiment 1A, the neuronal architectures of the GFP-labeled CA1 pyramidal neurons in the hippocampus were examined. The neuromorphological results are summarized in Table 2. Neither the male nor the female mice exhibited any significant genotype-dependent differences in GFP-labeled pyramidal neurons in the CA1 region of hippocampus in any of the morphological variables that we examined.

**Table 2:** **Neuromorphological analyses (mean ± s.e.m.) of GFP-labeled pyramidal neurons in the hippocampi of male and female TMc-*Nrg1*^+/−^ (*Nrg1*^+/−^) mice and their wild-type (WT) littermate controls**.

		**Male**		**Female**	
		**WT**	***Nrg1*^+/−^**	**WT**	***Nrg1*^+/−^**
Soma size (μm^2^)		92.090 ± 4.974	86.850 ± 5.725	86.270 ± 4.278	80.190 ± 6.040
Distance to apical bifurcation (μm)		241.100 ± 55.430	265.200 ± 66.730	257.100 ± 57.750	249.200 ± 65.130
Number of branches of apical branches		19.000 ± 2.781	18.170 ± 1.249	19.880 ± 2.356	19.500 ± 1.268
Number of apical tips		20.000 ± 2.781	18.830 ± 1.138	20.630 ± 2.412	20.380 ± 1.322
Total length of the apical tuft (μm)		3009.000 ± 397.700	2621.000 ± 465.900	2973.000 ± 370.100	2593.000 ± 348.000
Apical dendritic field area (ADFA) (× 1000 μm^2^)		11.100 ± 1.644	9.512 ± 2.588	10.850 ± 1.493	9.532 ± 1.980
Branch angle of primary apical dendrites (°)		49.270 ± 4.899	68.800 ± 13.010	69.650 ± 11.360	48.420 ± 7.560
Number of primary basal dendrites		3.444 ± 0.242	3.900 ± 0.315	3.286 ± 0.360	3.400 ± 0.221
The total length of primary basal dendrites (μm)		79.310 ± 16.820	87.610 ± 13.460	95.000 ± 30.090	69.120 ± 13.810
Number of branches of basal branches		20.110 ± 1.989	19.400 ± 0.957	22.430 ± 1.757	21.900 ± 1.980
Number of basal tips		22.780 ± 1.722	23.000 ± 1.000	25.000 ± 1.464	24.900 ± 1.894
The total length of basal dendrites (μm)		3301.000 ± 293.600	3001.000 ± 294.600	3590.000 ± 317.500	3179.000 ± 429.300
Basal dendritic field area (BDFA) (× 1000 μm^2^)		10.230 ± 1.309	8.047 ± 1.077	10.690 ± 1.284	10.720 ± 1.893
Sholl analysis of basal dendritic complexity					
Distance to soma (μm)	10	0.444 ± 0.342	1.900 ± 0.795	0.429 ± 0.578	1.300 ± 0.297
	20	4.222 ± 0.946	4.800 ± 0.712	3.571 ± 0.563	4.500 ± 0.429
	30	5.333 ± 1.108	7.100 ± 1.016	5.429 ± 0.674	7.100 ± 0.841
	40	7.333 ± 1.308	9.900 ± 1.394	10.140 ± 0.702	9.600 ± 0.829
	50	10.110 ± 0.955	12.400 ± 1.376	12.290 ± 0.605	11.900 ± 0.680
	60	12.000 ± 1.065	13.200 ± 1.083	13.570 ± 0.359	13.200 ± 0.841
	70	13.110 ± 0.872	14.500 ± 0.860	15.860 ± 0.716	14.300 ± 0.857
	80	14.110 ± 1.138	15.800 ± 0.554	17.000 ± 1.024	14.600 ± 0.951
	90	15.000 ± 1.065	16.400 ± 0.806	16.570 ± 1.016	15.100 ± 0.896
	100	14.780 ± 1.265	15.800 ± 0.827	16.570 ± 1.083	15.200 ± 1.088
	>100	9.127 ± 0.632	8.756 ± 0.725	9.108 ± 1.076	8.335 ± 0.509

#### Experiment 3B—examination of the expression of GABAergic markers in the hippocampus

**Figure 4:**
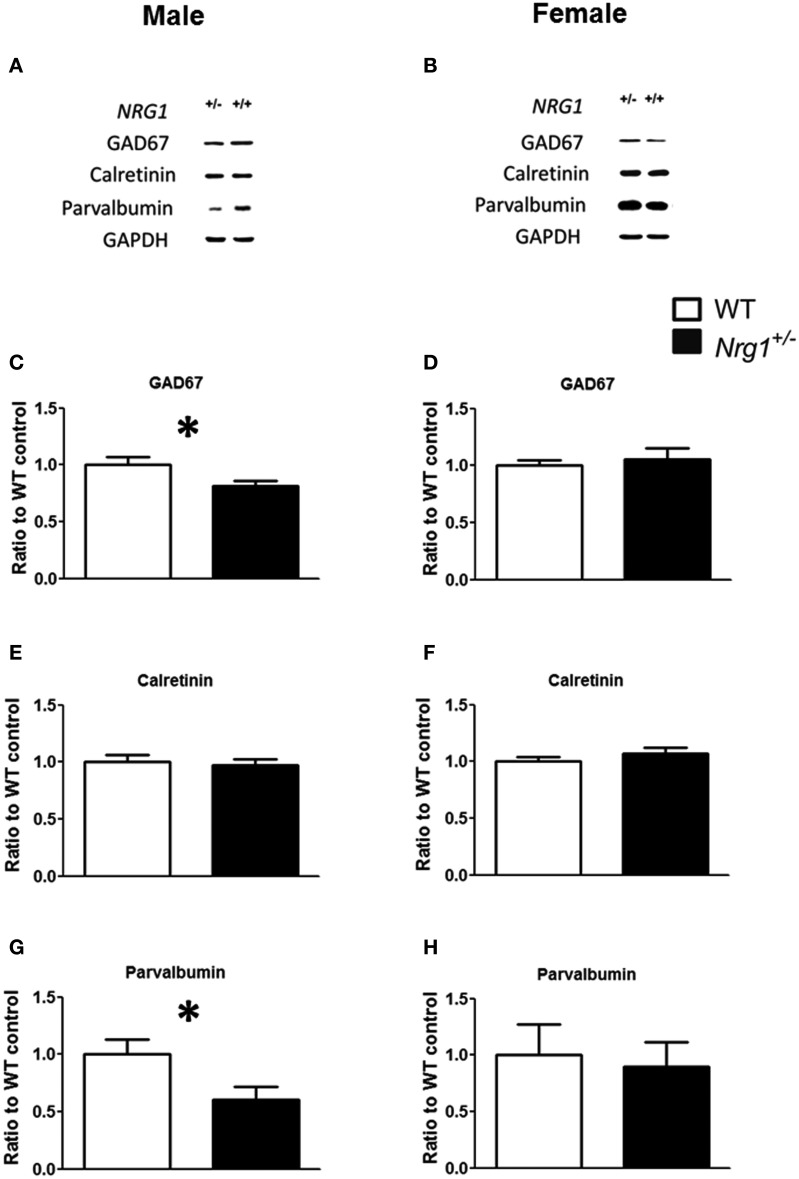
**The neurochemical analysis of the expression of GABAergic markers in the hippocampi of the novel TMc-*Nrg1*^+/−^mutant mice (black bars) and their wild-type controls (WT, white bars) determined by Western blot. (A,B)** Representative Western blots of hippocampal tissue (30 μg/lane) with GAD67 (67 kDa), calretinin (29 kDa), parvalbumin (12 kDa), and GAPDH (37 kDa) from males and females. **(C–H)** Quantification of the data revealed reductions in GAD67 and parvalbumin expression levels in the hippocampi of male **(C,E,G)**, but not female **(D,F,H)**, TMc-*Nrg1*^+/−^mice. Data are presented as the mean + s.e.m. ^*^P < 0.05 between two genotypes.

The protein expression levels of GAD67, parvalbumin (PV), and calretinin were examined in the hippocampi of both male and female mice. Representative images of protein expressions are shown in Figures 4A,B. Compared to WT controls, a significant reduction of GAD67 expression was found in our TMc-*Nrg1*^+/−^males [*t*_(12)_ = 2.245, *p* = 0.044; Figure 4C] but not in females (Figure 4D). Regarding calretinin, no genotype-dependent differences were found in either males or females (Figures 4E,F). Regarding parvalbumin, TMc-*Nrg1*^+/−^ males displayed a significant reduction of parvalbumin expression [*t*_(10)_ = 2.253, *p* = 0.048; Figure 4G] compared to WT controls, whereas the females did not exhibit this reduction (Figure 4H). The sex-specific reductions of hippocampal GAD67 and parvalbumin expression found in the TMc-*Nrg1*^+/−^ males of this experiment might be responsible for the observed cognitive deficits of these mice.

### Experiment 4: evaluation of the effect of valproate on the rescue of cognitive deficits in the novel TMc-*Nrg1*^+/−^ male mice

**Figure 5:**
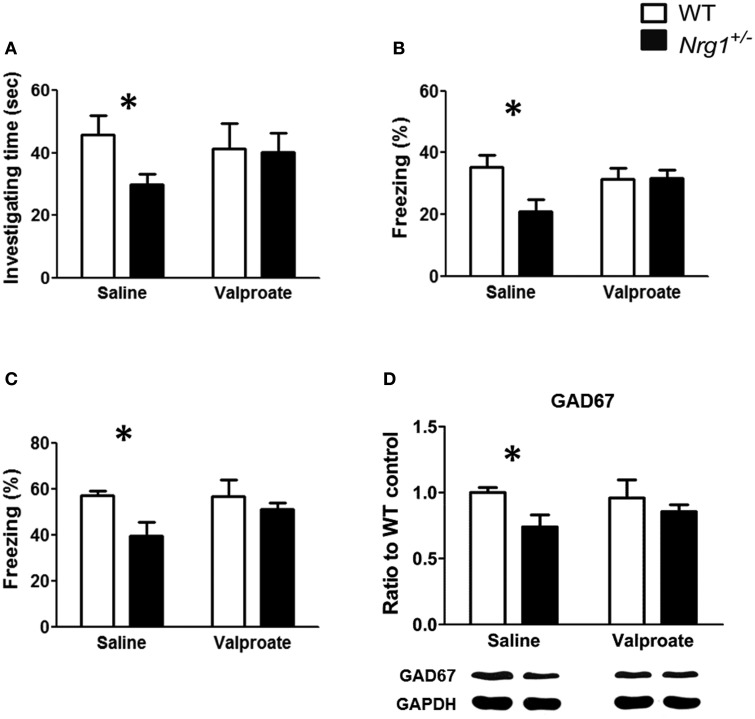
**The effect of chronic valproate treatment on the rescue of cognitive deficits in male wild-type (WT, white bars) and TMc-*Nrg1*^+/−^ mutant (black bars) mice**. Three cognitive tasks, including **(A)** an object recognition task, **(B)** a contextual fear conditioning task, and **(C)** a cued fear conditioning task, were conducted. Either valproate (1.5 mmol/kg, s.c.) or 0.9% saline was injected twice a day for 17 days. The object recognition task was conducted on day 16,and the contextual/cued tests of fear conditioning were performed on day 17. **(D)** The expression of GAD67 in the hippocampus of both WT and TMc-*Nrg1*^+/−^ mice after chronic injections of either saline or valproate. Data are presented as the mean + s.e.m. ^*^P < 0.05 between two genotypes.

Based on the findings of Experiments 1A, 2, and 3B, chronic administration of valproate was used to evaluate the effects of valproate on the rescue of the cognitive deficits that were observed in the male TMc-*Nrg1*^+/−^ mice. As reported in Experiment 1A, significant genotype-dependent reductions in behavioral performance on the object recognition task [*t*_(12)_ = 2.351, *p* = 0.037; Figure 5A], the contextual fear conditioning task [*t*_(12)_ = 2.556, *p* = 0.025; Figure 5B], and the cued fear conditioning task [*t*_(12)_ = 2.469, *p* = 0.029; Figure 5C] were found in the male TMc-*Nrg1*^+/−^mice that received chronic saline injections. In contrast, after chronic injections of valproate, male TMc-*Nrg1*^+/−^mice did not display any significant deficits in these tasks compared to WT control males (all *p* > 0.05; Figures 5A–C). For the expression of GAD67 in the hippocampus, a significant genotype-dependent reduction (~26%) of GAD67 was found in mice that received chronic saline treatment [*t*_(10)_ = 2.651; *p* = 0.024; Figure 5D]. In contrast, no significant reduction was found in the TMc-*Nrg1*^+/−^ male mice that received chronic valproate injections compared to their WT controls.

## Discussion

The use of genetically modified mice that carry a truncated TMc domain-*Nrg1* gene as an experimental tool offers an alternative model with which to mimic a NRG1 deficiency in some schizophrenic patients and to test a NRG1 loss-of-function hypothesis for schizophrenia. In complementary to the original TMc-Nrg1 mutant strain and other mutant strains, the impact of TMc exon ablation can be studied in both sexes using this novel TMc-*Nrg1*^+/−^ mouse strain and its functional consequences can be compared with findings in other Nrg1-related mouse strains. A significant reduction of Nrg1 proteins was also confirmed in the brain of this novel mutant strain. This novel TMc-*Nrg1*^+/−^ mouse strain provides a feasible model for the characterization of the roles of the TMc domain of *Nrg1* in basic behaviors and cognitive functions. In Experiment 1, we found that both male and female TMc-*Nrg1*^+/−^ mice displayed normal profiles of basic behaviors but were impaired in cognition-related functions. In Experiment 2, we found that, compared to WT controls, the injection of PTZ induced significant behavioral alterations in male (but not female) TMc-*Nrg1*^+/−^mice, whereas the other two drugs we tested had no effect. In Experiment 3, neuromorphological analyses failed to reveal any *Nrg1* genotype-dependent effects on the neuronal architecture of the GFP-labeled CA1 pyramidal neurons in the hippocampi of either male or female mice. However, reductions of GAD67 and parvalbumin expression were found in the hippocampi of male TMc-*Nrg1*^+/−^ mice, and females did not exhibit these reductions. In Experiment 4, the chronic administration of valproate successfully rescued the observed cognitive deficits of male TMc-*Nrg1*^+/−^ mice and hippocampal GAD67 expression.

To the best of our knowledge, this study is the first to characterize behavioral phenotypes of this novel TMc-*Nrg1*^+/−^ mutant strain. We also illustrated and verified that the truncated region had a hydrophobic protein structure that was located in the TMc of Nrg1. In complementary to those existing Nrg1-related mutant mouse strains, this novel mouse strain used in this study can be referred as a novel TMc-Nrg1 mutant strain. Our behavioral phenotyping data indicated that the truncation of the TMc domain in our TMc-Nrg1 mutant mice affected some cognitive functions but apparently did not have any effect on basic behaviors. The basic behavioral data reported in the present study, along with data regarding the basic behavioral phenotypes that have been reported for different lines of *Nrg1*-related mutant mice, are summarized in Table 3. There are several inconsistencies in this dataset; for example, PPI deficits have been observed in studies of type III Nrg1 mutant mice (Chen et al., 2008), *ErbB4* mutant mice (Shamir et al., 2012), ErbB2/4 mutant mice (Barros et al., 2009), and NRG1-overexpressing transgenic mice (Deakin et al., 2009; Kato et al., 2010), and in some (but not all) studies of the original TMc-*Nrg1* mutant mice (Stefansson et al., 2002; Desbonnet et al., 2012). These original TMc-*Nrg1*^+/−^ mutant mice have been widely used in recent years. It is of great interest to compare the behavioral phenotypes of these original TMc-Nrg1 mutant mice with those of our novel TMc-*Nrg1* mutant mice. As indicated in Table 3, the behavioral phenotypes of the original TMc-*Nrg1*^+/−^ mutant mice seem to vary somewhat across studies. Generally, this line of mutant mice has been reported to exhibit behavioral deficits in some behavioral tasks, such as the PPI, social recognition, and open field tasks (Stefansson et al., 2002; O'Tuathaigh et al., 2006, 2007; Boucher et al., 2007; Karl et al., 2007; Van Den Buuse et al., 2009; Chesworth et al., 2012; Desbonnet et al., 2012). However, our novel TMc-*Nrg1*^+/−^ mutant mice did not exhibit any significant impairment in these behavioral tasks. We also found no age-dependent effects in our novel TMc-Nrg1 mutant mice after examination at 2 and 6 months of age; this result is similar to reports that the age of onset is not associated with the NRG1 genotype in patients with schizophrenia (Kampman et al., 2004; Voineskos et al., 2009). In contrast, Karl et al. reported that the original TMc-*Nrg1*^+/−^ mutant mice do not display age-dependent hyperlocomotion until the age of 4.5 months (Karl et al., 2007), but these mice have been found to exhibit hyperlocomotion at 3 month of age in other studies (Van Den Buuse et al., 2009). Different targeting strategies were used to generate our new TMc-*Nrg1* mutant mice and the original TMc-*Nrg1* mutant mice. But as we described previously, it is possible that both TMc-*Nrg1* mutant mouse strains are targeting the same TMc exon due to differences in exon annotation. To further verify this possibility, we conducted a nucleotide BLAST search using our primer set and the forward primer sequence reported previously (Liu et al., 1998a; Lai et al., 2010). Based on the location of the forward primer and the size of PCR product, we found that the forward primer used for the original TMc-Nrg1 mutant mice located ~951 bps upstream of the forward primer used for our new TMc-Nrg1 mutant mice, suggesting both TMc-*Nrg1* mutant mouse strains are targeting the same exon. However, it should be noted that different targeting strategies can result in different biological functions. As far as we can tell from previous literatures (Liu et al., 1998a; Lai et al., 2010), the original TMc-*Nrg1* mutant strain was designed to generate most soluble forms of Nrg1 by replacing the TMc exon with a mini-exon that introduces stop codons right upstream of the TMc. In contrast, our new TMc-*Nrg1* mutant mice have no such added stop codons and are more likely to result in complete functional knockout of all Nrg1 isoforms except for those that naturally terminate before the TMc (such as SMDF). In addition to biological difference, abovementioned discrepancy could be due to different experimental designs, different experimental procedures (Van Den Buuse et al., 2009; Karl et al., 2011), age differences, or genetic background differences. As indicated in Table 3, differences in gene targeting strategy and the truncation of different domains or exon location in *Nrg1* gene appear to have differential effects on behavioral performance.

**Table 3:** **Summary of the behavioral phenotypes and comparison of the different lines of *Nrg1*-related mutant mice**.

**Genetic mouse model for**	**Behavioral phenotype**						
	**Learning and memory**	**Sensorimotor gating and information processing**	**Social**	**Motor function and locomotor activity**	**Explorative behavior**	**Anxiety-like behavior**	**Hedonia**
TM domain (exon 9, the novel TMc-Nrg1 mutant mice)	Object recognition ♂ ↓	PPI =	Preference =	OF =	HB =	EPM =	Sucrose =
	Contextual FC ♂ ↓		Recognition =	HB =	EPM =		
	Cued FC ♂ ↓						
	WMW =						
Background:							
C57BL/6							
TM domain (exon 11, the original TMc-Nrg1 mutant mice)	Object recognition ↓	PPI =	Preference =	OF ↑	Explorative behavior ↑	OF =	Sucrose =
	Contextual FC ↓	(Boucher et al., 2007)	Recognition ↓	(Stefansson et al., 2002)	♀ (O'Tuathaigh et al., 2006)	(Boucher et al., 2007)	(Desbonnet et al., 2012)
	Passive avoidance =	(Van Den Buuse et al., 2009)	(O'Tuathaigh et al., 2007)	♂ ♀ (O'Tuathaigh et al., 2006)		OF ↓ (PND138; 60)	
	(Duffy et al., 2010)	PPI ↓	(Desbonnet et al., 2012)	(Boucher et al., 2007)	HB ↑	(Karl et al., 2007)	
Background:	♀ (Chesworth et al., 2012)	(Stefansson et al., 2002)	Interaction =	PND 138	LD ↑ (vertical activity)	(Desbonnet et al., 2012)	
C57BL/6	Cued FC =	(Desbonnet et al., 2012)	(Boucher et al., 2007)	(Karl et al., 2007)	(Boucher et al., 2007)	EPM ↓	
	(Duffy et al., 2010)		(Desbonnet et al., 2012)	15-week-old	(Karl et al., 2007)	LD ↑	
	Cued FC ↓	Startle =	Aggression ↑	♀ (Chesworth et al., 2012)	LD =	(Boucher et al., 2007)	
	♀ (Chesworth et al., 2012)	(Boucher et al., 2007)	♂ ♀ (O'Tuathaigh et al., 2007)	3-month-old	(Karl et al., 2007)		
	Radial arm maze =	Startle ↓	(O'Tuathaigh et al., 2008)	(Van Den Buuse et al., 2009)		EPM =	
	(Duffy et al., 2010)	(Desbonnet et al., 2012)	Aggression =	OF =		LD =	
	Y-maze =		Agonistic behavior ↑	PND 91		(Karl et al., 2007)	
	♂ ♀ (O'Tuathaigh et al., 2007)		(Desbonnet et al., 2012)	(Karl et al., 2007)		(O'Tuathaigh et al., 2008)	
	(Duffy et al., 2010)		Agonistic behavior =	7–8-month-old			
	♀ (Desbonnet et al., 2012)		(Boucher et al., 2007)	(Van Den Buuse et al., 2009)			
	Barnes maze ↓		(O'Tuathaigh et al., 2008)				
	♂ (O'Tuathaigh et al., 2007)						
	Cheeseboard =						
	♀ (Chesworth et al., 2012)						
EGF-like Domain (exon 6)	Contextual FC ↓	PPI =	Recognition ↓	OF ↑	HB =	OF ↓	
	Object recognition =	(Duffy et al., 2008)	(Ehrlichman et al., 2009)	LD ↑	(Duffy et al., 2008)	EPM =	
	(Ehrlichman et al., 2009)	(Ehrlichman et al., 2009)		Marble burying =		LD =	
Background:							
C57BL/6							
(Duffy et al., 2008)							
	Cross maze =	MMN ↓		(Duffy et al., 2008)		(Duffy et al., 2008)	
	(Duffy et al., 2008)	P20 =		OF =			
		P40 =		(Ehrlichman et al., 2009)			
		(Ehrlichman et al., 2009)		Bar test =			
C57BL/6 × 129/SVEV				Rotard rod =			
				(Michailov et al., 2004)			
(Ehrlichman et al., 2009)							
Ig-like domain (exon 3)	T maze =			Running wheel =			
	Latent inhibition ↓ (Rimer et al., 2005)			(Rimer et al., 2005)			
Background:							
C57BL/6							
Type III	DNMS ↓	PPI ↓		OF =			
Background:	(Chen et al., 2008)	(Chen et al., 2008)		(Chen et al., 2008)			
C57BL/6							
ErbB4^+/−^		PPI =		OF ↑			
Background:		(Stefansson et al., 2002)		(Stefansson et al., 2002)			
C57BL/6							
CNS-specific ErbB4 KO	MWM			OF ↓ delayed motor development (Golub et al., 2004)			
	♂ ↓ (Golub et al., 2004)						
Background:							
C57BL/6							
Heart-rescued ErbB4 KO	Cued FC ↓	PPI ↓		OF ↓		EPM ↓	
	Contextual FC ↓	(Shamir et al., 2012)		(Shamir et al., 2012)		(Shamir et al., 2012)	
	(Shamir et al., 2012)						
Background:							
C57BL/6							
PV-Cre; ErbB4	Cued FC =	PPI ↓		OF ↑		EPM =	
	Contextual FC =	(Shamir et al., 2012)		(Shamir et al., 2012)		(Shamir et al., 2012)	
Background:	(Shamir et al., 2012)						
C57BL/6 × 129/SVEV	Radial arm maze ↓						
	(Wen et al., 2010)						
CNS-specific							
ErbB2/4 KO							
Background:							
FVB		PPI ↓	Aggressive ↑			OF ↓	
		(Barros et al., 2009)	(Barros et al., 2009)			(Barros et al., 2009)	
Transgenic NRG1	Contextual FC ↓	PPI ↓	Interaction ↑	OF ↑ (Kato et al., 2010)			
	Cued FC =	(Kato et al., 2010)	Aggression ↑				
Background: not described	(Kato et al., 2010)		(Kato et al., 2010)				
Transgenic	Y-maze = (11 month)	%PPI ↓		OF = (5, 7.5, 10 month)			
Type I	DNMS = (3 month)	ASR ↑		OF ↑ (12.5 month)			
Background:	DNMS ↓ (10 month)	(Deakin et al., 2012)		(Deakin et al., 2012)			
C57BL/6	(Deakin et al., 2012)			Rotard rod =			
				(Michailov et al., 2004)			
				Rotard rod ↓			
				Nesting ↓			
				Burrowing ↓			
				(Deakin et al., 2009)			

A comparison of the behavioral phenotypes of different mutants (left column, from top to bottom), including the novel TMc- Nrg1^+/−^ mutant mice, the original TMc-Nrg1^+/−^ mutant mice, Nrg1 heterozygous knockout mice with an EFG-like domain truncation of exon 6, Nrg1 heterozygous knockout mice with an Ig-like domain truncation (type I and II) of exon 3, Type III (CRD-domain) Nrg1 mutant mice, ErbB4 mutant mice, CNS-specific ErbB4 knock-out mice, ErbB4 knock-out mice that were rescued from embryonic lethality by reexpression of ErbB4 in the heart, parvalbumin-positive-specific ErbB4 knock-out mice, CNS-specific ErbB2/4 knock-out mice, Nrg1 overexpression transgenic mice, and type I Nrg1 overexpression transgenic mice. All studies summarized in this table were conducted in only male Nrg1-related mice except one study used only female mice and two studies used both male and female mice. Among these 3 studies, ♂ indicates that only male (but not female) mutants showed behavioral deficits, and ♀ indicates that only female (but not male) mutants showed behavioral impairments. Behavioral phenotypes are divided based on tasks (top row, from left to right) into categories that include learning and memory, sensorimotor gating, information processing, social function, motor function and locomotor activity, explorative behavior, anxiety-like behavior, and hedonia. NORT, novel object recognition task; FC, fear conditioning task; MWM, Morris water maze; DNMS, delay non-match to sample tasks; PPI, prepulse inhibition; ASR, auditory brain response; startle, startle response; MMN, mismatch negativity; P20, P20 event-related potential (ERP); P40, P40 ERP; OF, open field task; LD, light-dark chamber task; HB, hole broad task; EPM, elevated plus maze task; sucrose, sucrose preference task; ↑, increased in mutant mice compared to wild type littermates; ↓, decreased in mutant mice compared to wild type littermates; =, no difference between mutant mice and wild type littermates.

A sex-specific effect of Nrg1 on cognitive function was found in Experiment 1 of this study. Our male TMc-*Nrg1*^+/−^ mutant mice exhibited behavioral deficits in the novel object recognition task, contextual fear conditioning task, and cued fear conditioning task, whereas females did not display these impairments. As indicated in Table 3, many *Nrg1*-related studies have only been conducted on male mice. Interestingly, either haploinsufficiency of Nrg1 or overexpression of Nrg1 (Deakin et al., 2009, 2012; Kato et al., 2010) in mice can result in some types of cognitive deficits, which implies a possible U-shaped relationship between Nrg1 expression and cognitive function, which has been reported for the *COMT* gene (Honea et al., 2009). Few studies have examined female mice, and these studies have reported that the original TMc-*Nrg1*^+/−^ mutant female mice exhibit hyperlocomotion, altered social recognition, and impaired contextual and cued fear conditioning (O'Tuathaigh et al., 2006, 2007; Chesworth et al., 2012). In contrast, both male and female TMc-*Nrg1*^+/−^ mutants and their WT littermates were examined in this study. The basic and cognitive functions of our novel TMc-*Nrg1*^+/−^ mutant female mice seemed to be largely unaffected when compared to their female WT controls. The sex-specific alterations of cognitive function observed in our male TMc-*Nrg1*^+/−^mutant mice are somewhat consistent with the gender-specific differences in patients with schizophrenia (Kulkarni et al., 2012). This finding also suggests a potential interaction between NRG1/ErbB signaling and sex hormones (e.g., estrogen) in the regulation of schizophrenia-related cognitive deficits; a similar interaction has been described in human studies (Wong and Weickert, 2009; Agim et al., 2013). Examination of the protective effects of estrogen (i.e., the estrogen protection hypothesis of schizophrenia) in our TMc-*Nrg1* mutant mice is warranted.

In Experiment 2, based upon major neurotransmitter-based hypothesis of schizophrenia, 3 related drugs were chosen to evaluate drug-induced behavioral alterations in our novel TMc-*Nrg1*^+/−^mutant mice. Our behavioral data revealed a sex-specific effect of PTZ (but not MK-801 or methamphetamine) on PTZ-induced responses in male TMc-*Nrg1*^+/−^mutant mice. It has been reported that Nrg1 interacts with NMDA receptors through Fyn kinase and Pyk2 (proline-rich tyrosine kinase 2) and that attenuated Nrg1 signaling affects the phosphorylation of NR2B receptors in *Nrg1*^+/−^mutant mice with either TMc-domain truncation or EGF-like domain truncation (Bjarnadottir et al., 2007). Moreover, subchronic injections of MK-801 (0.2 mg/kg, s.c.) disrupt sociability and social novelty preference in the original TMc-*Nrg1*^+/−^mutant mice (O'Tuathaigh et al., 2010). However, compared with WT controls, different doses of MK-801 (0.01, 0.05, 0.25 mg/kg, i.p.) did not affect MK-801-induced hyperlocomotion in the original TMc-*Nrg1*^+/−^ mutant mice (Van Den Buuse et al., 2009), which is consistent with our current findings in the new TMc-*Nrg1*^+/−^mutant mice. Additionally, no significant differences were found in [^3^H]MK-801 and [^3^H]kainate binding levels with Nrg1 status in the brains of the original TMc-*Nrg1*^+/−^ mutant mice using *in situ* radioligand binding (Dean et al., 2008). Glutamate and GABA concentrations in the brains of these original TMc-*Nrg1*^+/−^ mutant mice also did not differ from those of WT mice (O'Tuathaigh et al., 2010). In contrast, alteration of GABAergic neurons is considered as one of the most reliable abnormalities found in post-mortem analyses of the schizophrenic brain (Lewis et al., 2005). Accumulating evidence emerging from human genetic studies suggests the involvement of GABA-A receptor subunit genes in schizophrenia (Lewis et al., 2003) and the genetic link between GAD67 and early-onset schizophrenia (Addington et al., 2005). Dysfunction of cerebral cortex (especially PFC) and hippocampus in schizophrenia is also thought to include alteration in GABAergic, inhibitory neurotransmission (Guidotti et al., 2005; Lewis et al., 2005). Although PTZ is usually considered as a convulsant drug and has less face validity to schizophrenia compared to MK-801 and methamphetamine, the well-established PTZ-induced responses appear to be a useful index for evaluating the vulnerability of GABAergic system in mutant mice with reasonable construct validity and predictive validity. Our data suggest that the GABA transmission of our male TMc-*Nrg1*^+/−^ mutant mice was affected to a greater extent than glutamatergic and dopaminergic transmission. The truncation of different loci of Nrg1 might have differential effects compared to other Nrg1-related mutant mice.

Furthermore, because both Nrg1 and ErbB4 are highly abundant in the hippocampus (Corfas et al., 1995; Law et al., 2004; Vullhorst et al., 2009) and also because some hippocampus-dependent cognitive deficits were observed in Experiment 1, we examined GFP-labeled CA1 pyramidal neurons to elucidate any neuromorphological alterations of excitatory neurons in the hippocampus. This study might be the first to analyze morphometric alterations of CA1 pyramidal neurons in TMc-related *Nrg1*^+/−^ mutant mice. However, no significant genotype-dependent neuromorphological alterations were found in either the males or females in any of the morphological variables we examined, suggesting that the neuromorphology and function of these hippocampal excitatory units may be intact in our TMc-*Nrg1*^+/−^mutant mice. Our current results suggest that the GABAergic system is affected to a greater extent than either the glutamatergic or dopaminergic systems in the brains of our mutant mice, especially the males. It has been proposed that NRG1 has both forward and reverse functions in ErbB/NRG1 signaling. The forward signaling via ErbBs promotes the formation of the excitatory and inhibitory synapses of interneurons. The reverse signaling is ErbBs-independent; Nrg1 is cleaved by gamma-secretase to release the intracellular domain (NRG1-ICD), which is important for the development of cortical pyramidal neurons (Chen et al., 2008, 2010; Fazzari et al., 2010; Pedrique and Fazzari, 2010). Although more work needs to be performed to elucidate the role of NRG1-ICD in the hippocampus, our current data suggest that the deficiency of TMc domain might have a minor impact on reverse signaling that alters the neuromorphology of CA1 pyramidal neurons, whereas this haploinsufficiency might have a major impact on the development and neuroplasticity of interneuron that is mediated through forward signaling; these suppositions are also supported by the PTZ-induced behavioral alterations we observed in our TMc-*Nrg1*^+/−^ mutant mice.

Additionally, further evidence arose from Experiment 3B, in which we found sex-specific reductions on GAD67 and parvalbumin expression in the hippocampi of our TMc-*Nrg1*^+/−^ mutant mice. Indeed, it has been reported that Nrg1 and ErbB4 signaling controls the development of inhibitory circuitries in the mammalian cerebral cortex through GABAergic interneurons (Barros et al., 2009; Vullhorst et al., 2009; Pedrique and Fazzari, 2010; Wen et al., 2010; Cahill et al., 2012) and that Nrg1 treatment has a synaptogenic effect, which is possibly mediated by the stabilization of PSD-95 on GABAergic interneurons, but not glutamatergic neurons (Ting et al., 2011). A genetic association between ErbB4 and human cortical GABA levels has also been reported (Marenco et al., 2011). Besides, exogenous NRG1 down-regulated the expression of GABA_A_ receptors in hippocampal CA1 pyramidal neurons (Okada and Corfas, 2004). Our findings from Experiment 3B are concordant with these findings and indicate the importance of Nrg1 in the regulation of GAD67 and parvalbumin expression in the hippocampus. Thus, haploinsufficiency of Nrg1 resulted in reductions in hippocampal GAD67 and parvalbumin expression, which may have caused alterations in the inhibitory interneuron networks by desynchronizing gamma oscillations [as has been proposed previously (Gonzalez-Burgos and Lewis, 2008; Del Pino et al., 2013)] and caused the cognitive impairments we observed in male Nrg1 mutant mice. This mechanism may contribute to the pathogenesis of some cognitive deficits in some patients with schizophrenia and *Nrg1* alterations.

The effect of valproate on the rescue of the cognitive deficits observed in our male TMc-*Nrg1* mutant mice should be of great interest to researchers. Valproate is a pharmacoepigenomic agent that has epigenetic effects on the modification of GABAergic interneurons (Csoka and Szyf, 2009). Valproate has been reported to ameliorate cognitive impairments in adult mice via demethylation of GABAergic-promotors (Tremolizzo et al., 2002, 2005; Matrisciano et al., 2013), inhibition of histone deacetylases (Phiel et al., 2001), and enhancement of central GABAergic tone that is mediated through an inhibition of GABA-transaminase (Johannessen, 2000). The reciprocal interaction between defects of NRG1 and hypermethylation of GABAergic promotors remains unclear. Our findings indicate that deficiencies of Nrg1 resulted in reductions of hippocampal GAD67 and parvalbumin expression, which might affect hippocampus-related cognitive functions. Interestingly, protracted treatment with valproate ameliorated observed cognitive deficits and the reduction of hippocampal GAD67 expression in our male TMc-*Nrg1* mutant mice. It has also been reported that protracted valproate treatment increased GAD67 mRNA expression in the brain, which likely facilitated GABAergic neurotransmission (Loscher, 1999; Tremolizzo et al., 2002, 2005). Notably, in terms of the pharmacokinetics of valproate, the dose we used has been shown to be comparable to the effective concentrations (0.15–0.30 mmol/kg) of valproate that are administered to psychiatric patients (Tremolizzo et al., 2005). Accordingly, the observed cognitive deficits that resulted from the haploinsufficiency of Nrg1 and the reduction of GABAergic transmission in our male TMc-*Nrg1* mutant mice were likely to be ameliorated by the upregulation of the GABAergic system due to chronic valproate injections. It is of interest to confirm hippocampal GAD67 and parvalbumin expressions in a new batch of valproate-treated Nrg1 mutant mice that did not receive any behavioral testing and further examine its epigenetic effect. Although the underlying mechanisms require further investigation, our data suggest that valproate has great potential for improving cognitive deficits in patients with schizophrenia, especially males with NRG1 haploinsufficiency. Further studies are greatly needed.

### Conflict of interest statement

The authors declare that the research was conducted in the absence of any commercial or financial relationships that could be construed as a potential conflict of interest.
