# Supplementary material for: Distinct phenotypes of new transmembrane-domain neuregulin 1 mutant mice and the rescue effects of valproate on the observed schizophrenia-related cognitive deficits
Source: Front Behav Neurosci. 2014 Apr 14;8:126. doi: 10.3389/fnbeh.2014.00126 (PMC3995064; doi:10.3389/fnbeh.2014.00126)
Supplement: Supplementary file 1 [file Presentation1.PDF]

Distinct phenotypes of new transmembrane-domain neuregulin 1 mutant mice and the rescue effects of valproate on the observed schizophrenia-related cognitive deficits.

Ju-Chun Pei [1], Chih-Min Liu [2], Wen-Sung Lai * [1, 3, 4]

[1] Department of Psychology, National Taiwan University, Taipei, Taiwan

[2] Department of Psychiatry, National Taiwan University Hospital, Taipei, Taiwan

[3] Graduate Institute of Brain and Mind Sciences, National Taiwan University, Taipei, Taiwan

[4] Neurobiology and Cognitive Science Center, National Taiwan University, Taipei, Taiwan

*** Correspondence:** Wen-Sung Lai, Department of Psychology, National Taiwan University, 1 Roosevelt Road, Sec. 4, Taipei 10617, Taiwan

wslai@ntu.edu.tw

Keywords: schizophrenia, transmembrane-domain Nrg1 mutant mice, cognitive function, hippocampus, GABAergic interneuron, valproate.

Supplementary Materials

Experiment 1: Behavioral testing procedures

Two series of experiments were conducted to examine the effects of Nrg1 deficiency on the cognitive functions (Experiment 1A) and basic behaviors (Experiment 1B) of these mice. The cognitive tasks included an object recognition task, a fear conditioning task, the Morris water maze task, and a prepulse inhibition task; these tasks are thought to mainly measure hippocampal-dependent behaviors. Basic behaviors were evaluated with a comprehensive battery of behavioral tasks that consisted of an open-field locomotor assay, a hole board task, an elevated plus maze task, a social preference and recognition task, a sucrose preference task, and a hot plate task. The first cohort of mice (n = 12 each) was sequentially tested with the novel object recognition task on postnatal days 63-65 (P63-65), with the fear conditioning task on P66-70 and with the Morris water maze on P90-110. The other cohort of mice (male = 12; female = 10) was tested with the open field task on P63 and P189, with the hole board task on P65 and P191, the elevated plus maze task on P67 and P193, the social preference and recognition task on P71, the sucrose preference task on P72-75 and P199-202, and the prepulse inhibition task on P78. The general principle of this arrangement was to avoid performing more stressful tasks prior to less stressful tasks to minimize carryover effects.

Novel object recognition task: Mice have an innate tendency to prefer to explore novel objects over familiar objects. Two objects with different shapes, colors and textures were used in this task, and the preference for each object was balanced. The task consisted of three trials, including habituation, training, and testing trials, with a 15-min inter-trial interval between each trial. One day before the test, each subject was acclimated to a polyvinylchloride chamber (25 cm x 24 cm x 25 cm) for 10 minutes. On the testing day, each subject was allowed to explore the same cage freely for a 5-min habituation trial. In the training trial, each mouse was presented with a pair of identical objects for 10 minutes. In the testing trial, one object was replaced by a novel object, and each mouse was allowed to explore for another 10 minutes. The whole experiment was videotaped by digital cameras, and the times spent investigating each object were recorded using ETHOM software (Shih and Mok, 2000). Investigative behavior was defined by forelimb contact or sniffing of the object.

Contextual and cued fear conditioning: This task was conducted using a commercially available fear-conditioning system (TruScan 2.01 system, Coulbourn Instruments, Allentown, PA, U.S.A.). On the training day, animals were first introduced into the conditioning chamber for a 3-min free exploration period. Two tone-foot shock pairings (tone (CS), 30 sec; foot shock, 2 sec, 0.8 mA, termination with the tone) that were separated by a 1-min intertrial interval occurred. A one-min post-shock free exploration period occurred before each mouse was returned to its home cage. On the second day, contextual fear conditioning was tested in the same chamber for 6 minutes. One day later, each mouse was introduced into a novel chamber with different environmental cues (different visual cues and a gray plastic floor) for the auditory cued fear conditioning test. After a 3-min baseline period (pre-CS), three 3-min tones (CS) separated by 1-min intertrial intervals were delivered. All behaviors were videotaped by digital cameras, and total freezing time, defined as a complete lack of movement excluding respiration, was recorded using ETHOM software.

Morris water maze: Spatial learning and memory abilities were examined with a standard Morris water maze. A circular pool (diameter, 100 cm) that was filled with water, which had been clouded with a nontoxic white paint and was kept at 22 ± 1 °C, was placed in the center of the testing room and surrounding surrounded with several visual cues. A platform (12×12 cm^2) was hidden 1 cm beneath the surface of the water. The swim paths of each subject were recorded using an EthoVision tracking system (Noldus Information Technology, Wageningen, Netherlands). Each mouse was given 4 trials per day with intertrial intervals of 10-15 minutes for 8 consecutive training days. On each daily trial, each mouse started from a quadrant that was pseudorandomly selected from among the three quadrants that did not contain the platform. Mice that failed to find the platform within 1 min were gently guided to the platform, where they remained there for 30 sec before being returned to their cages. Escape latencies (sec), measured as the time required to find the hidden platform, and path lengths (cm) were recorded. After an 8-day acquisition training, a 1-day retention test was performed by removing the platform and placing each subject back into the

pool for 1 min. The times spent swimming and the swimming distances in each quadrant were recorded and used as an index of reference memory ability.

Pre-pulse inhibition (PPI): To assess sensorimotor gating function, each subject was tested in the SR-LAB startle apparatus (San Diego Instruments, San Diego, CA, USA). The background noise was 72 dB during testing. Beginning with a 5 min acclimatization period followed by 64 trials, each session could be dividing into four blocks. The first and last blocks were composed of 6 trials of a 120 dB pulse alone. The second and third blocks consisted of 52 trials with 5 types of stimuli that were selected pseudorandomly and separated by inter-trial intervals that averaged 15 sec. The 5 types of stimuli were as follows: (1) a 40-ms, 120-dB white noise burst (pulse-alone); (2–4) 120-dB startle stimuli that were preceded by 100 ms with a 20-ms noise burst of either 78-, 82- or 90-dB (pre-pulse); and (5) background noise only. PPI percentages (PPI %) were calculated as 100 x [(pulse-alone − PPI)/pulse-alone], where the pulse-alone score was taken as the average of the pulse-alone values from the second and third blocks of 52 trials. The pulse-alone responses of the first and last blocks were taken as the indices of the startle responses and habituation of the startle responses, respectively.

Social preference and social recognition task: This task was conducted in a 48 cm x 24 cm x 25 cm arena that was equally divided into two zones. Two transparent polyethylene terephthalate bottles (diameter, 6.5 cm; height, 13 cm) with holes (diameter of 0.6 cm) in the walls were used to restrain the stimulus mouse to facilitate social interact while preventing direct physical contact; these bottles were placed in the centers of the two zones. The testing procedure consisted of five habituation trials and one social recognition trial with a 5-min intertrial interval between each trial. Each subject was first introduced into the arena with two empty bottles for a 5-min habituation period. After a 5-min intertrial interval, each subject was placed back to the arena for five 5-min habituation trials in which a stimulus mouse (stranger #1) was restrained in one of the bottles, and the other bottle remained empty. The first habituation trial was used to evaluate the social preference of each mouse. The percentage of social preference was calculated as 100 x (time spent in the zone with stimulus mouse / total time). After 5 habituation trials, the social recognition trial was conducted to measure social recognition in these mice. A 5-min social recognition trial was conducted by introducing a novel stimulus mouse (stranger #2) into the previously empty bottle, and the times spent in either zone were measured. The percentage of social recognition was calculated as 100 x (time spent in the zone with a novel mouse / total time). All stimulus mice were the same age and gender as the subjects. The location of the stimulus mice in the two bottles was counterbalanced to avoid place preference effects. The duration of time each subject spent in each zone was recorded and analyzed using an EthoVision video tracking system (Noldus Information Technology, Netherlands).

Open field task: To measure spontaneous locomotor activity and anxiety-like behavior, each subject was placed into a polyvinylchloride chamber (48 cm x 24 cm x 25 cm) for 60 minutes. Total

travel distances, travel distances in 5-min, and aversive ratios were recorded using an EthoVision video tracking system (Noldus Information Technology, Netherlands). Hyperactivity was defined as a lack of habituation to the novel environment, and anxiety-like behavior can be considered to be relevant to the psychomotor agitation and mood symptoms of patients with schizophrenia.

Hole board task: A 16-hole (diameter of 2.2 cm) board (25.2 cm x 25.2 cm x 2.4 cm) in an open-field apparatus (25.40 cm x 25.40 cm x 40.64 cm) with infrared photo sensors (TruScan 2.01 System, Coulbourn Instruments, Whitehall, PA, USA) was used to evaluate explorative behavior over a 30-minute period. Total and 5-min bin head dipping frequencies were automatically recorded and used as indices of symptoms of excitement and psychomotor agitation.

Elevated plus maze task: A standard white Plexiglas elevated-plus maze at a height of 50 cm above the floor was used to evaluate anxiety levels over a 5-min period. The apparatus comprised two open arms and two closed arms (50 cm × 10 cm × 50 cm) that extended from the center of the maze. The duration spent in each arm was automatically recorded and analyzed with an EthoVision video tracking system (Noldus Information Technology, Netherlands). The numbers of head dippings, rearings (i.e., when the forelimbs left the floor in the open arms) and risk assessments (i.e., head sniffing in the open arms while the body was located at the closed arm) were also recorded by a well-trained observer. The aversive ratios and frequencies of head dippings, rearings and risk assessments were used as additional indices for anxiety.

Sucrose preference: A two-bottle sucrose preference test was used to evaluate the reward sensitivity of our mutant mice. Each subject was individually tested in its home cages. The two bottles were filled with diluent (drinking water) on the first day to obtain a 24-h drinking baseline. Subsequently, bottles were filled with 1% and 2% sucrose solutions on days 2 and 3. Daily fluid intakes were measured by weighing the bottles, and the positions of the bottles were alternated daily. Daily sucrose preferences were calculated for each mouse as follows: 100 × [2% sucrose fluid intake / (1% sucrose fluid intake + 2% sucrose fluid intake)]. Decreases in the preference for the 2% sucrose solution were used as an index of anhedonia.
